# Supplementary material for: Mitochondrial Transfer from Human Platelets to Rat Dental Pulp-Derived Fibroblasts in the 2D In Vitro System: Additional Implication in PRP Therapy
Source: Int J Mol Sci. 2025 Jun 8;26(12):5504. doi: 10.3390/ijms26125504 (PMC12192669; doi:10.3390/ijms26125504)
Supplement: Supplementary file 1 [file ijms-26-05504-s001.zip › captions of supplemental figures.pdf]

VIDEO S1. Time-course changes in resting platelets in co-culture with RPC-C2A cells. Living human platelets in the resting state were added to rat fibroblasts and cultured for 25 h. The initial one hour was used for platelet precipitation, and video recording was started one hour after platelet addition and continued for 24 h.

VIDEO S2. Time-course changes in activated platelets in co-culture with RPC-C2A cells. Living human platelets in the resting state were added to rat fibroblasts and cultured in the presence of ADP for 25 h. Time-lapse video recording was continued for 24 h as described in the legend of Video S1.

Supplemental figures

Figure S1. (A) Effects of ADP on CD62P expression, a marker of activated platelets, in cultured platelets. Platelets were cultured in the absence or presence of 3 μM ADP for 8 h. At the end of the culture, platelets were probed with FITC-conjugated anti-CD62P monoclonal antibody (1:20) (BioLegend, San Diego, CA, USA) for 60 min at 20-23°C. Platelets were then gently washed and fixed with 10% formalin for 10 min. The CD62P expression was examined by a fluorescence microscope. Scale Bar = 10 μm. **(B)** Effects of ADP on platelet aggregation. After appropriate amounts of $CaCl_2$ were added, platelets suspended in the plasma were stimulated with 3 μM ADP at 37°C using an aggregometer (PRP3000S, TAIYO, Osaka, Japan), as described previously [52]. The three traces drawn by the software provided by the manufacturer represent the simultaneous assay of the sample in triplicate.

Figure S2. Visual images of platelets and RPC-C2A cells, each alone or in combination. Platelets suspended in the medium were cultured alone (A) or with RPC-C2A cells (C) for 12 h. For reference, RCP-C2A cells cultured alone were examined (B). ADP-added co-cultures were also examined to visualize the possible changes in platelet adhesion (D). Scale bar = 200 μm (low mag.) or 100 μm (high mag.)
