# Supplementary material for: Mitochondrial Transfer from Human Platelets to Rat Dental Pulp-Derived Fibroblasts in the 2D In Vitro System: Additional Implication in PRP Therapy
Source: Int J Mol Sci. 2025 Jun 8;26(12):5504. doi: 10.3390/ijms26125504 (PMC12192669; doi:10.3390/ijms26125504)
Supplement: Supplementary file 1 [file ijms-26-05504-s001.zip › Supple_data (Figs).pptx]

## Slide 1
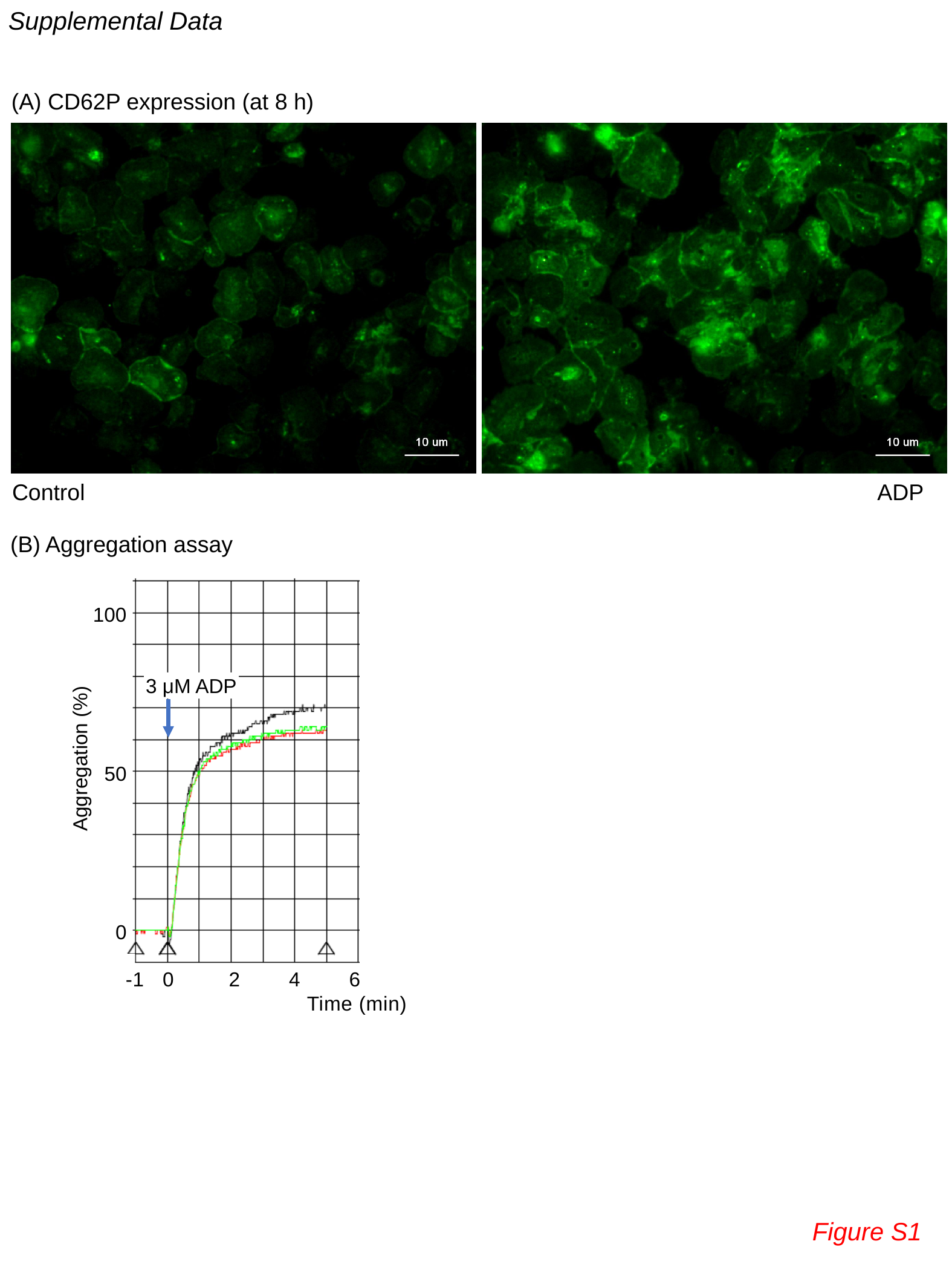

Supplemental Data
(A) CD62P expression (at 8 h)
Control 									 ADP
(B) Aggregation assay
100
50
0
3 μM ADP
Aggregation (%)
-1 0 2 4 6
		Time (min)
Figure S1

## Slide 2
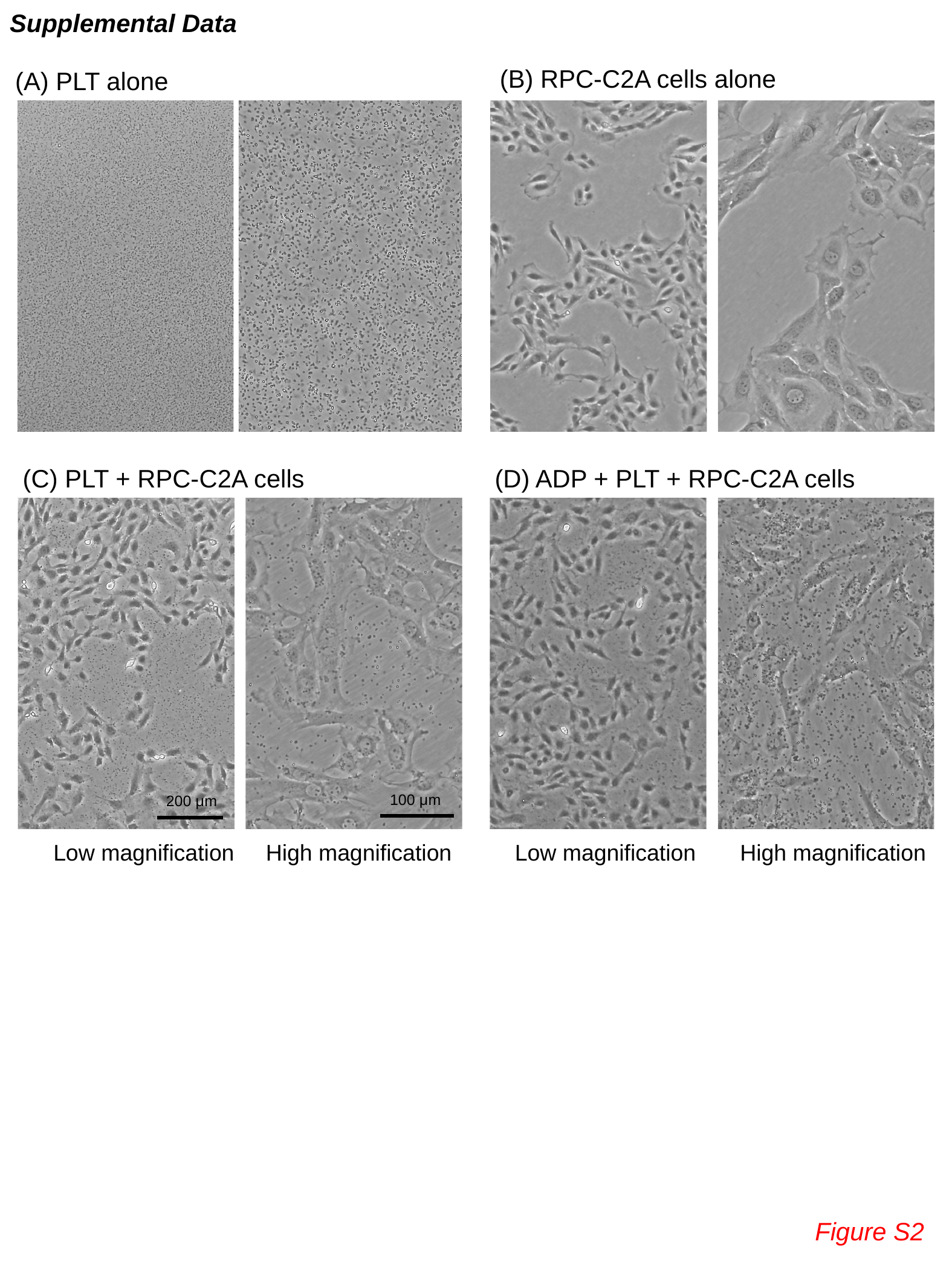

Supplemental Data
(B) RPC-C2A cells alone
(A) PLT alone
(C) PLT + RPC-C2A cells
(D) ADP + PLT + RPC-C2A cells
100 μm
200 μm
Low magnification High magnification Low magnification High magnification
Figure S2
